# Epigenetic down-regulation of the *HIST1* locus predicts better prognosis in acute myeloid leukemia with *NPM1* mutation

**DOI:** 10.1186/s13148-019-0738-6

**Published:** 2019-10-12

**Authors:** Sylvain Garciaz, Lia N’guyen Dasi, Pascal Finetti, Christine Chevalier, Julien Vernerey, Mathilde Poplineau, Nadine Platet, Stéphane Audebert, Matthieu Pophillat, Luc Camoin, François Bertucci, Boris Calmels, Christian Récher, Daniel Birnbaum, Christian Chabannon, Norbert Vey, Estelle Duprez

**Affiliations:** 1 Epigenetic Factors in Normal and Malignant Hematopoiesis Team, Aix Marseille University, CNRS, Inserm, Institut Paoli-Calmettes, CRCM, 27 Boulevard Lei Roure, 13273 Marseille Cedex 09, France; 2 Predictive Oncology Laboratory, CRCM, Inserm, U1068, CNRS UMR7258, Institut Paoli-Calmettes, Aix-Marseille University, Marseille, France; 3 Institut Pasteur, G5 Chromatin and Infection, Paris, France; 4 Aix-Marseille University, Inserm, CNRS, Institut Paoli-Calmettes, CRCM, Marseille Protéomique, Marseille, France; 5 Aix-Marseille University, Inserm, CNRS, Institut Paoli-Calmettes, CRCM, Centre d’Investigations Cliniques en Biothérapies, Marseille, France; 6 Service d’Hématologie, Centre Hospitalier Universitaire de Toulouse, Institut Universitaire du Cancer de Toulouse Oncopole, Toulouse, France Université Toulouse III Paul Sabatier, Cancer Research Center of Toulouse, UMR1037-INSERM, ERL5294 CNRS, Toulouse, France; 7 Aix-Marseille University, Inserm, CNRS, Institut Paoli-Calmettes, CRCM, Marseille, France

**Keywords:** Epigenetics, H3K27me3, Acute myeloid leukemia, *HIST1*, *NPM1*

## Abstract

**Background:**

The epigenetic machinery is frequently altered in acute myeloid leukemia. Focusing on cytogenetically normal (CN) AML, we previously described an abnormal H3K27me3 enrichment covering 70 kb on the *HIST1* cluster (6.p22) in CN-AML patient blasts. Here, we further investigate the molecular, functional, and prognosis significance of this epigenetic alteration named H3K27me3 *HIST1* in *NPM1*-mutated (*NPM1*mut) CN-AML.

**Results:**

We found that three quarter of the *NPM1*mut CN-AML patients were H3K27me3 *HIST1*^high^. H3K27me3 *HIST1*^high^ group of patients was associated with a favorable outcome independently of known molecular risk factors. In gene expression profiling, the H3K27me3 *HIST1*^high^ mark was associated with lower expression of the histone genes *HIST1H1D*, *HIST1H2BG*, *HIST1H2AE*, and *HIST1H3F* and an upregulation of genes involved in myelomonocytic differentiation. Mass spectrometry analyses confirmed that the linker histone protein H1d, but not the other histone H1 subtypes, was downregulated in the H3K27me3 *HIST1*^high^ group of patients. H1d knockdown primed ATRA-mediated differentiation of OCI-AML3 and U937 AML cell lines, as assessed on CD11b/CD11c markers, morphological and gene expression analyses.

**Conclusions:**

Our data suggest that *NPM1*mut AML prognosis depends on the epigenetic silencing of the *HIST1* cluster and that, among the H3K27me3 silenced histone genes, *HIST1H1D* plays a role in AML blast differentiation.

**Graphical abstract:**

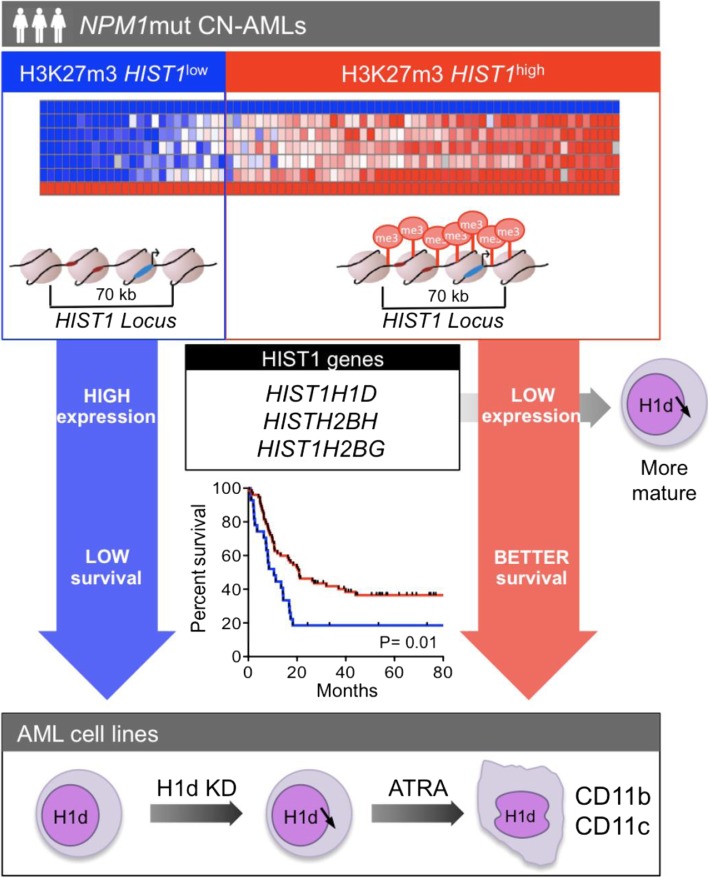

**Electronic supplementary material:**

The online version of this article (10.1186/s13148-019-0738-6) contains supplementary material, which is available to authorized users.

## Background

Acute myeloid leukemias (AMLs) are a heterogeneous group of severe hematological malignancies that arise through the acquisition of oncogenic mutations by hematopoietic progenitor cells. Patient prognosis mainly relies on the given treatment, the outcome of which depends on cytogenetics and molecular alterations. Cytogenetically normal (CN) AML patients are usually assigned to an intermediate prognosis group that can be further subdivided through the detection of mutations in a growing number of genes [1]. Mutations in the nucleophosmin 1 (*NPM1*) gene are the commonest molecular lesions occurring in ≥ 50% of cases with cytogenetically normal acute myeloid leukemia (CN-AML). *NPM1* mutations result in the generation of a nuclear export signal causing the delocalization of the protein from the nucleoli to the cytoplasm [2]. Analyses of large numbers of patients have shown that *NPM1* mutations are associated with a relatively favorable prognosis, which can be mitigated by internal tandem duplications (ITD) of the tyrosine kinase receptor Fms-like tyrosine kinase 3 (*FLT3*) and mutations in DNA-methyl transferase 3A (*DNMT3A*). Indeed, in *NPM1*mut CN-AML, *FLT3*ITD mutation, and/or mutation in *DNMT3A* predict an increased risk of relapse and poorer outcome [3], [4]. Recent reports shed light on the importance of epigenetic deregulations that affect the epigenome and gene transcription in AML pathogenesis [5]. These deregulations are the consequence of numerous alterations found in genes encoding multiple classes of epigenetic proteins as DNA methylation and histone modification enzymes [6].

In addition to these mutations, recent reports underlined the importance of histone genes themselves in cancer onset. For example, the major histone cluster 1 (*HIST1*), encoding the vast majority of the redundant core and linker histones [7], has been found partially deleted in ALL [8] and deregulation of its expression is associated with breast cancer invasiveness [9]. Moreover, we previously described a new alteration that affects the *HIST1* cluster in AML. This alteration consists in a marked histone H3 lysine 27 tri-methylation (H3K27me3) enrichment encompassing 70 kb of the *HIST1* cluster, affecting histone genes and associates with the *NPM1* mutation and a better leukemia-free survival [10]. In this study, we reported the clinical importance of this newly identified epigenetic alteration, called the H3K27me3 *HIST1* mark, in relation to other known mutations and its functional consequences on the biology of CN-AML leukemic cells. We revealed that H3K27me3 *HIST1* status and histone mRNA and protein levels define clinically and biologically different subgroups of NPM1mut CN-AML suggesting their importance in AML pathogenesis.

## Results

### H3K27me3 level on *HIST1* locus is associated with better survival

**Fig. 1:**
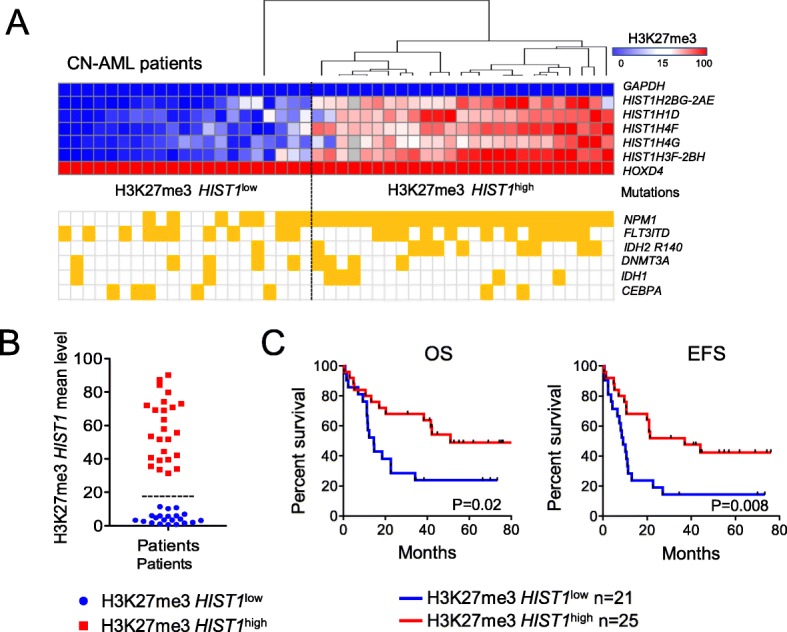
Analysis of H3K27me3 *HIST1* level in CN-AML patients. **a** Heatmap of normalized H3K27me3 enrichment value obtained by H3K27me3 ChIP-qPCR on the indicated *HIST1* genes. Enrichment was calculated as the percentage of bound/input and double normalized with *HOXD4* and *GAPDH*. Each column represents a patient sample (*n* = 46) sorted by unsupervised hierarchical clustering. The separation between H3K27me3 *HIST1*^low^ and H3K27me3 *HIST1*^high^ groups is indicated by a dotted lane. Yellow square: mutation for the indicated gene. **b** Bimodal distribution of the patients according to H3K27me3 *HIST1* mean level, obtained from the H3K27me3 enrichment values of the 5 *HIST1* regions analyzed. In blue, patients with an H3K27me3 *HIST1* mean value below 15 (H3K27me3 *HIST1*^low^, *n* = 21), in red, patients with a mean value above 15 (H3K27me3 *HIST1*^high^, *n* = 25). **c** OS and EFS in CN-AML patients according to H3K27me3 *HIST1*^low^ and H3K27me3 *HIST1*^high^ status

To characterize the H3K27me3 *HIST1* mark, we performed H3K27me3 ChIP-qPCR on samples obtained from 46 de novo CN-AML patients included in GOELAMS multicenter clinical trials LAM2006IR (NCT00860639) or LAM2007SA (NCT00590837). H3K27me3 level was determined at five *HIST1* genomic locations that are representative of the H3K27me3 *HIST1* signature, as described previously [10]. Heatmap showing H3K27me3 *HIST1* gene enrichment confirmed the variation of H3K27me3 *HIST1* level among CN-AML patients (Fig. 1a). The average of the five normalized H3K27me3 *HIST1* values was calculated and this index showed a clear segregation of the H3K27me3 *HIST1*^low^ and H3K27me3 *HIST1*^high^ patients. With a cut-off value at 15, approximately 55% of CN-AML samples displayed an H3K27me3 *HIST1* enrichment mark (Fig. 1b).

There was no association of H3K27me3 *HIST1* status with age, gender, *FLT3*ITD, *DNMT3A*, *IDH1*, or CCAAT/enhancer-binding protein alpha (*CEBPA*) mutations (Fig. 1a and Additional file 1: Table S1). However, we noted that 25/33 (75.7%) of the *NPM1*mut samples were also H3K27me3 *HIST1*^high^ (Fig. 1a and Additional file 1: Table S1) confirming our first observation [10]. In addition, we observed a significant association between H3K27me3 *HIST1*^high^ and the presence of *IDH2*R140 mutation (36% vs. 4.7%, *P* = .01) (Fig. 1a and Additional file 1: Table S1).

H3K27me3 *HIST1*^high^ patients had a better overall survival (OS) and event-free survival (EFS) than H3K27me3 *HIST1*^*low*^ patients, with a median OS of 50.9 months versus 14.6 months (HR, 2.5 [1.5–5.5]; *P* = .02) and a median EFS of 37 months versus 9 months (HR, 2.7 [1.3–5.8]; *P* = .008) (Fig. 1c). The survival gain was independent in multivariate analyses taking age and *NPM1*wt/*FLT3*ITD status into account (Additional file 1: Table S2). These observations confirmed in an independent cohort the previously reported association of H3K27me3 *HIST1*^high^ with a better prognosis [10].

### H3K27me3 level on *HIST1* locus is an independent biomarker predicting survival of NPM1mut CN-AML

**Fig. 2:**
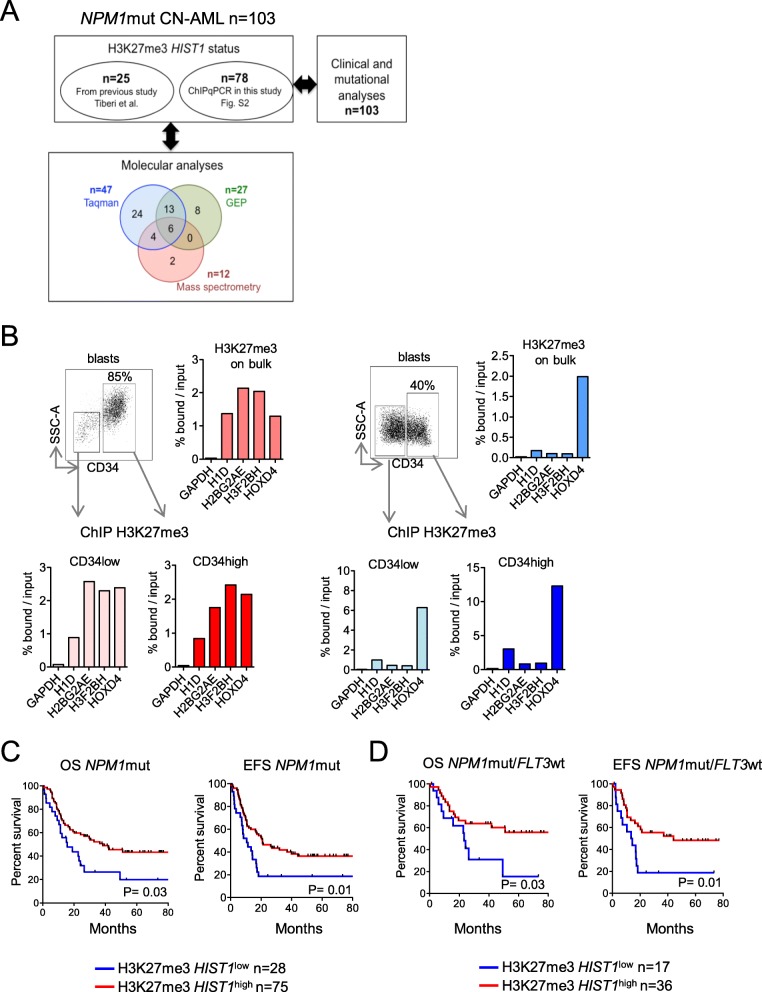
Analysis of H3K27me3 *HIST1* level in CN-AML patients with *NPM1* mutation. **a** Consort diagram showing number and overlap of *NPM1*mut AML samples analyzed in the different experiments. **b** Analysis of H3K27me3 *HIST1* status in CD34low and CD34high blast populations. The left panel presents an H3K27me3 *HIST1*^high^ patient and the right panel an H3K27me3 *HIST1*^low^ patient. For each panel are shown: the gating strategy for separating blast cells according CD34 expression level (upper left), the H3K27me3 *HIST1* status on the bulk population (upper right), on CD34low blast population (lower left) and on CD34high blast population (lower right). **c** OS and EFS in *NPM1*mut CN-AML patients according to H3K27me3 *HIST1* status (*n* = 103). **d** OS and EFS in *NPM1*mut/*FLT3*wt CN-AML patients according to H3K27me3 *HIST1* status (*n* = 53). Statistical significance was estimated using the log-rank test

*NPM1*-mutated AMLs represent a distinct clinical and biological entity in the World Health Organization (WHO) classification, commonly associated with a better prognosis [3]. We used the biological material obtained from 25 *NPM1*mut samples previously analyzed [10], and 78 samples either provided by the GOELAMSTHEQUE (*n* = 33), or the IPC/CRCM tumor bank (*n* = 45) (Fig. 2a). H3K27me3 status of the 78 new samples was analyzed (Additional file 2: Figure S1) and revealed that 75% of *NPM1*mut CN-AMLs were H3K27me3 *HIST1*^high^. The *NPM1*mut H3K27me3 *HIST1*^high^ subgroup of patients was not enriched with *DNMT3A* or *FLT3*ITD mutations, the most frequently *NPM*1mut co-occurring alterations [11] (Table 1 and Additional file 2: Figure S1), but *IDH2*R140 was significantly overrepresented in this subgroup in comparison with the *NPM1*mut H3K27me3 *HIST1*^low^ group (27.6% vs. 7.6%, *P* = .05). Interestingly, H3K27me3 *HIST1*^high^ leukemic cells had a significantly lower CD34 expression than their H3K27me3 *HIST1*^low^ counterparts (CD34 mean expression, 10.3% vs. 35%, *P* = 0.005) (Table 1). To further explore a potential association between CD34 expression and the presence of H3K27me3 *HIST1* mark, we selected four patient samples (two in each H3K27me3 *HIST1* group), containing both CD34low and CD34high blast populations. We next analyzed the H3K27me3 *HIST1* level in CD34low and CD34high fluorescence-activated cell sorted (FACS) blasts. As shown in Fig. 2b and Additional file 2: Figure S2, H3K27me3 *HIST1* status (high or low), in both CD34^low^ and CD34^high^ blast fractions, was similar to the bulk population, indicating that H3K27me3 *HIST1*^high^ occurs independently of CD34 level of expression.

**Table 1:** Clinical and molecular characteristics according to H3K27me3 *HIST1* level in the *NPM1*mut AML cohort

Characteristics	All patients (*n* = 103)	H3K27me3 *HIST1*^low^ (*n* = 28)	H3K27me3 *HIST1*^high^ (*n* = 75)	*P*
Age, years				0.42
Median	61	62	60	
Range	22–76	37–76	22–76	
Sex, %				1.0
Male	43	42.8	44	
WBC, × 10e9/L	67	87.9	55.6	0.57
Median	67	87.9	55.6	
Range	10–352	10–352	11–230	
Complete response, %	94	88.4	96.0	0.18
Allo-HSCT in 1st CR, %	23	32.1	20.0	0.2
FAB classification,%				1.0
0–2	51.8	50.0	52.6	
4–5	48.2	50.0	47.4	
CD34 expression^a^				0.005
Mean	15.3	35.0	10.3	
Range	0–99	12–99	0–93	
Molecular alterations, %				
*FLT3*ITD	47.0	37.0	50.6	0.36
*DNMT3A*^b^	50.5	57.6	43.1	0.25
*FLT3*ITD/DNMT3A^b^	23.1	23.1	23.1	1.0
*IDH2* (R140)^b^	21.9	7.6	27.6	0.05
*IDH1* (R132)^b^	12.1	15.3	10.7	0.72
*CEBPA*^b^	5.4	7.6	4.6	0.62
*ASXL1*^b^	3.2	3.8	3.1	1.0

*Allo HSCT* allogenic stem-cell transplantation, *CR* complete response, *WBC* white blood cell

^a^*N* = 72

^b^*N* = 91

Next, we analyzed the prognosis impact of the H3K27me3 *HIST1* alteration. *NPM1*mut H3K27me3 *HIST1*^high^ patients had a better OS and EFS than *NPM1*mut H3K27me3 *HIST1*^*low*^ patients (median OS, 38.3 vs. 15.7 months; HR, 2 [range, 1.0–3.0]; *P* = .03; median EFS, 20.9 vs. 10.6 months; HR, 2.7 [range, 1.3–5.7]; *P* = .01) (Fig. 2c). In multivariate analysis, taking age and *FLT3*ITD/*DNMT3A*mut into account, H3K27me3 *HIST1*^high^ remained significantly associated with a favorable EFS suggesting that the prognostic significance of the H3K27me3 *HIST1* signature is independent of other known molecular alterations (Table 2).

**Table 2:** Multivariate analyses for H3K27me3 *HIST1* status

	Event-free survival		
	Multivariate analysis		
Variables	HR	95% CI	*P*
H3K27me3 *HIST1*^high^	1.76	1.04–2.99	0.036
Age > 60 years	0.60	0.36–0.99	0.044
*FLT3*ITD/*DNMT3* mut	0.48	0.27–0.83	0.009

Finally, we studied the impact of H3K27me3 *HIST1* mark in the *NPM1*mut/*FLT3*wt subgroup, which is classically associated with a favorable prognosis [12]. H3K27me3 *HIST1*^high^ patients (*n* = 36) displayed better OS and EFS than H3K27me *HIST1*^low^ patients (*n* = 17), (median OS, 111.6 months vs. 23.2 months; *P* = .03; median EFS, 44.1 months vs. 13.9 months; *P* = .01, for H3K27me3 *HIST1*^high^ and H3K27me3 *HIST1*^low^, respectively) (Fig. 2d).

Our results suggest that H3K27me3 *HIST1* status is an independent epigenetic marker that identifies patients with a poor outcome within the *NPM1/FLT3*wt group of patients.

### Histone mRNA expression is anti-correlated to H3K27me3 *HIST1* level and predicts *NPM1*mut CN-AML patient outcome

**Fig. 3:**
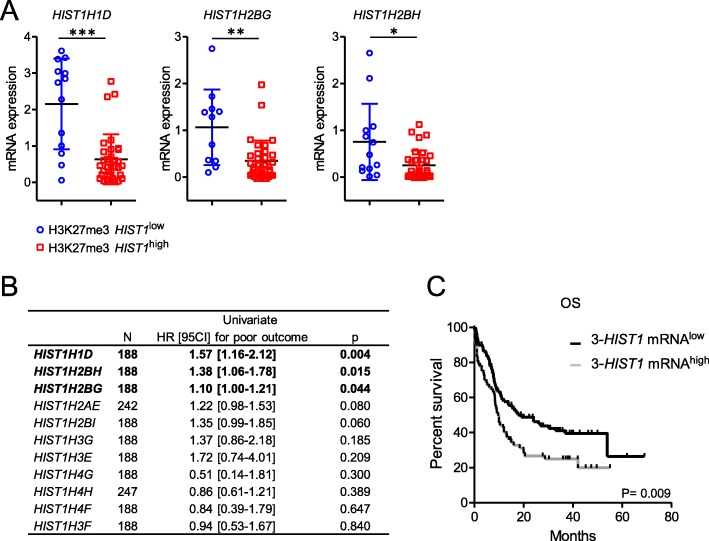
Gene expression from the *HIST1* cluster impacts on survival in *NPM1*mut CN-AML. **a** mRNA analyses of *HIST1* genes in the IPC cohort of *NPM1*mut CN-AML patients. *HIST1H1D* (*n* = 46), *HIST1H2BH* (*n* = 45), and *HIST1H2BG* (*n* = 47) mRNA levels were determined by Taqman, according to H3K27me3 *HIST1* status. Gene expression was normalized on the average of two housekeeping genes (*PGK1* and *PPIA*). Statistical significance was estimated using Mann Whitney test * *p* < 0.05; **p < 0.005 ***p < 0.0001. **b** Influence of histone gene expression on OS by univariate analysis. Only histone genes covered by the H3K27me3 *HIST1* mark described in [10] were analyzed. **c** Survival analyzed according to the expression of *HIST1H1D*, *HIST1H2BG*, and *HIST1H2BH* mRNA on independent cohorts of patients (TGCA and Metzeler). Patients were split into two groups according to the expression of *HIST1H1D*, *HIST1H2BH* and *HIST1H2BG* genes using a Cox regression prognosis model. Statistical significance was estimated using the log-rank test

To analyze the anti-correlation of histone mRNA level and the presence of the repressive H3K27me3 mark, we selected three histone genes; *HIST1H1D*, *HIST1H2BG* and *HIST1H2BH*, spread over the H3K27me3 *HIST1* islet and associated with clinical outcome in public data (see below), and measured their mRNA levels. Expression of these three genes was lower in H3K27me3 *HIST1*^high^ patients (*n* = 34) than in H3K27me3 *HIST1*^low^ patients (*n* = 13) (Fig. 3a).

We next asked whether expression of these genes, as a consequence of H3K27me3 mark, was associated with patient survival. Given the small size of our cohort, we analyzed *HIST1* gene expression in two published cohorts with publicly accessible clinical and mRNA expression data: TCGA [13] and Metzeler [14]. *NPM1*mut CN-AML patients, within these two cohorts, were identified by using a published gene expression signature that predicts the *NPM1* mutational status [15] (see Additional file 3: Supplemental methods). Association of histone expression with survival was first tested for each of the 11 histone genes covered by the H3K27me3 *HIST1* mark. This highlighted three histone genes, *HIST1H1D*, *HIST1H2BG*, and *HIST1H2BH*, for which a high level of expression was associated with a poor outcome (*P* = .004, .015 and .044 respectively, Fig. 3b). Then, we tested this 3-*HIST1*-mRNA signature in univariate analysis; 3-*HIST1*-mRNA^low^ patients had a favorable OS with a median OS of 17.7 months versus 9.6 months (HR = 1.66, range, 1.13–2.42, *P* = .009) (Fig. 3c). Multivariate analyses showed that the 3-*HIST1*-mRNA^low^ status was associated with a better prognosis (HR = 1.60, range 1.60–2.31, *P* = .01), independently of other prognosis markers including age, FAB classification and *FLT3* status (Additional file 1: Table S3).

These results show that H3K27me3 *HIST1*^high^ is associated with a lower expression of histone genes, and that 3-*HIST1*-mRNA^low^ signature defines a *NPM1*mut AML patient group with a better outcome.

### Gene expression profiling associated with H3K27me3 *HIST1*^high^ identifies a “mature like” phenotype

**Fig. 4:**
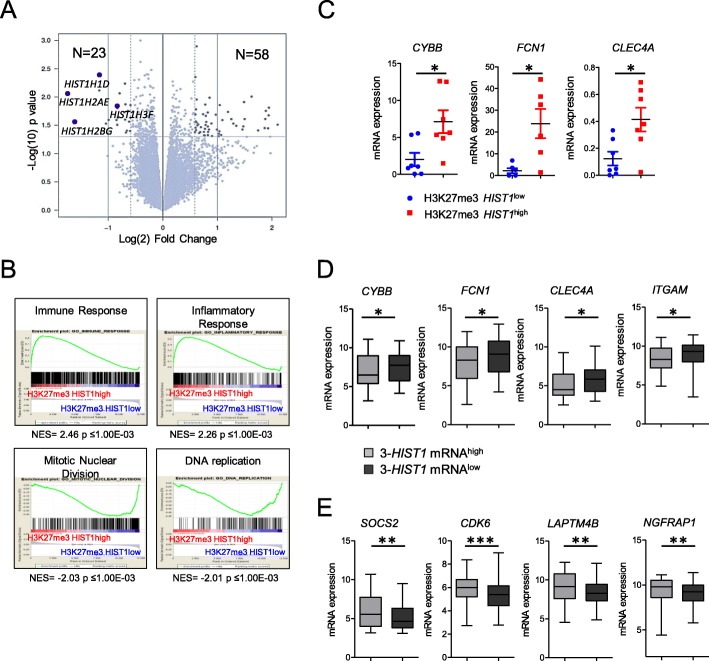
H3K27me3 *HIST1*^high^ is associated with a myelomonocytic Gene Expression Profile. **a** Volcano plot displaying differentially expressed genes between H3K27me3 *HIST1*^high^ (*n* = 16) and H3K27me3 *HIST1*^low^ patients (*n* = 11). The blue dots represent the histone genes covered by the H3K27me3 mark. **b** *EZH2* and *SUZ12* levels of expression obtained from our micro array data were analyzed in H3K27me3 *HIST1*^low^ (*n* = 11) and H3K27me3 *HIST1*^high^ (*n* = 16) patients. **c** GSEA (gene set enrichment analysis) of H3K27me3 *HIST1*^high^ samples. **d** Expression of three genes associated with granulocytic functions according to H3K27me3 *HIST1* status. *CYBB* (cytochrome B-245 beta chain); *FCN1* (Ficolin 1); *CLEC4A* (C-type lectin domain family 4 member A). Data are represented in relative expression to *HPRT.* **e**–**f** Patients from TGCA and GSE 61804 cohorts were separated according to the 3-*HIST1* mRNA signature in mRNA^low^ (*n* = 114) and mRNA^high^ (*n* = 79) patients. **e** Myelomonocytic *CYBB*, *FCN1*, *CLEC4A*, *ITGAM* (integrin subunit alpha M) and **f** leukemic stem cell *SOCS2* (suppressor of cytokine signaling 2), *CDK6* (cyclin dependent kinase 6), *LAPTM4B* (lysosome-associated protein transmembrane-4β), and *NGFRAP1* (nerve growth factor receptor-associated protein 1) gene expression was analyzed. Statistical significance was estimated using Mann Whitney test * *p* < 0.05; **p < 0.005 ***p < 0.0001. *NS* non-significant

We next characterized the gene expression profile (GEP) of H3K27me3 *HIST1*^high^ samples (*n* = 16) in comparison to H3K27me3 *HIST1*^low^ samples (*n* = 11) from the IPC cohort (see Additional File 1: Table S4 for patient clinical characteristics). Eighty-one genes were differentially expressed (*p* < .05, fold-change > 1.5) between the two groups, 58 being up- and 23 being downregulated in the H3K27me *HIST*1^high^ group (Fig. 4a and Additional file 4: Table S5). Analysis of enhancer of zeste homolog 2 (*EZH2*) and suppressor of zeste 12 (*SUZ12*) expression revealed that the two groups, H3K27me3 *HIST1*^high^ and H3K27me3 *HIST1*^low^, equally expressed PRC2 components (Fig. 4b). GSEA identified, in H3K27me3 *HIST1*^high^ patients, genes associated with myelomonocytic differentiation such as immune or inflammatory responses (Fig. 4c and Additional file 4: Table S5). Downregulated genes in these patients belong to cell cycle and chromatin regulation categories, including histone genes from the *HIST1* cluster (Fig. 4c and Additional file 4: Table S5). Using qPCR, we confirmed higher expression of three genes involved, in mature granulocyte functions, *CYBB*, *FCN1*, and *CLEC4A* [16], [17], [18] in H3K27me3 *HIST1*^high^ patients (Fig. 4d). H3K27me3 level at the promoter of these genes was identical between H3K27me3 *HIST1*^low^ and H3K27me3 *HIST1*^high^ patients (Additional file 2: Figure S3) suggesting that the variation of expression was indirectly affected by H3K27me3 *HIST1*^high^.

To further validate the relation between low mRNA level of *HIST1* genes and the expression of granulocytic markers, we tested mRNA expression of myelomonocytic maturation genes (*CYBB*, *FCN1*, *CLEC4*, and *ITGAM*) in the TCGA and Metzeler cohorts of patients stratified with the previously defined 3-*HIST1*-mRNA signature. The 3-*HIST1*-mRNA^low^ patient group overexpressed the differentiation genes in comparison to the 3-*HIST1*-mRNA^high^ one (Fig. 4e), thus corroborating our previous observation (Fig. 4d). Reciprocally, genes such as *SOCS2*, *CDK6*, *LAPTM4B*, and *NGFRAP1*, which were described as associated with a leukemic stem cell signature [19], were less expressed in the 3-*HIST1*-mRNA^low^ patient group (Fig. 4f).

Taken together, these results suggest that *HIST1* mRNA downregulation by the H3K27me3 *HIST1*^high^ mark is associated with a more differentiated phenotype related to a committed state of leukemic cells.

### The histone linker H1-3 is poorly expressed in H3K27me3 *HIST1*^high^ CN-AML patients

**Fig. 5:**
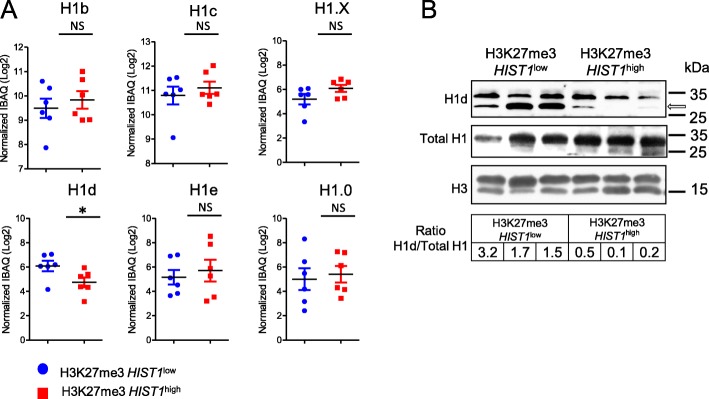
H1-3 protein expression is lower in H3K27me3 *HIST1*^*high*^ patients. **a** Abundance of histone H1 subtypes. Results of each H1 subtype are expressed in iBAQ (Log2) adjusted on the median of the total protein extracted and normalized to total histone amount by patients. Statistical significance was estimated using unpaired *t* test, (*) *p* < 0.05. *NS* non significant. **b** Immunoblots of H3, H1, and H1-3 on patient chromatin bound extracts. The arrow points to the specific band for H1-3. Ratio of H1-3/total-H1 is calculated using Mean Signal Intensity on imageJ software

To study the role of histones on AML clinical and biological features, we looked at the effect of H3K27me3 *HIST1* epigenetic silencing on histone protein level. First, we looked at the proportions of total histones and of each histone subtype (Additional file 2: Figure S4) in chromatin-bound fractions extracted from a series of 12 patient samples (six in each group) using intensity-based absolute quantification (iBAQ) approach. Normalized quantities of total linker histone H1 and core histones H2A, H2B, H3, and H4 were similar in both H3K27me3 *HIST1*^high^ and H3K27me3 *HIST1*^low^ patients (Additional file 2: Figure S5), suggesting that H3K27me3 *HIST1*^high^ status did not globally modify histone protein abundance*.* Then, we decided to analyze specifically the histone linker H1-3, encoded by *HIST1H1D*, because its mRNA level is affected by H3K27me3 *HIST1* status (Fig. 3a) and it is the leading gene for the mRNA signature (Fig. 3b). In addition, contrary to other histone subtypes (i.e., H2A, H2B, H3, and H4), H1 histone subtypes are heterogeneous in amino acid composition [20], which probably reflects a subtype-specific function. Indeed, when looking at the H1 subtype abundance, we observed that the H1-3 subtype was decreased in the H3K27me3 *HIST1*^high^ group (normalized iBAQ value (Log2) = 6.09 vs 4.74; *P* = .04), whereas the other H1 subtypes, H1-5, H1-2, H1-4, H1-F0, and H1-FX, were unaffected (Fig. 5a). These results are consistent with the specific *HIST1H1D* mRNA expression decrease observed in AML samples harboring the H3K27me3 *HIST1* mark. We confirmed the lower expression of H1-3 observed in H3K27me3 *HIST1*^high^ group in comparison to H3K27me3 *HIST1*^low^ group of patients by Western blot using pan H1 and specific H1-3 antibodies (Fig. 5b).

In conclusion, as a consequence of the presence of an H3K27me3 islet, *NPM1*mut CN-AML H3K27me3 *HIST1*^high^ patients express low level of H1-3.

### H1-3 knockdown confers a more mature phenotype in AML cell lines

**Fig. 6:**
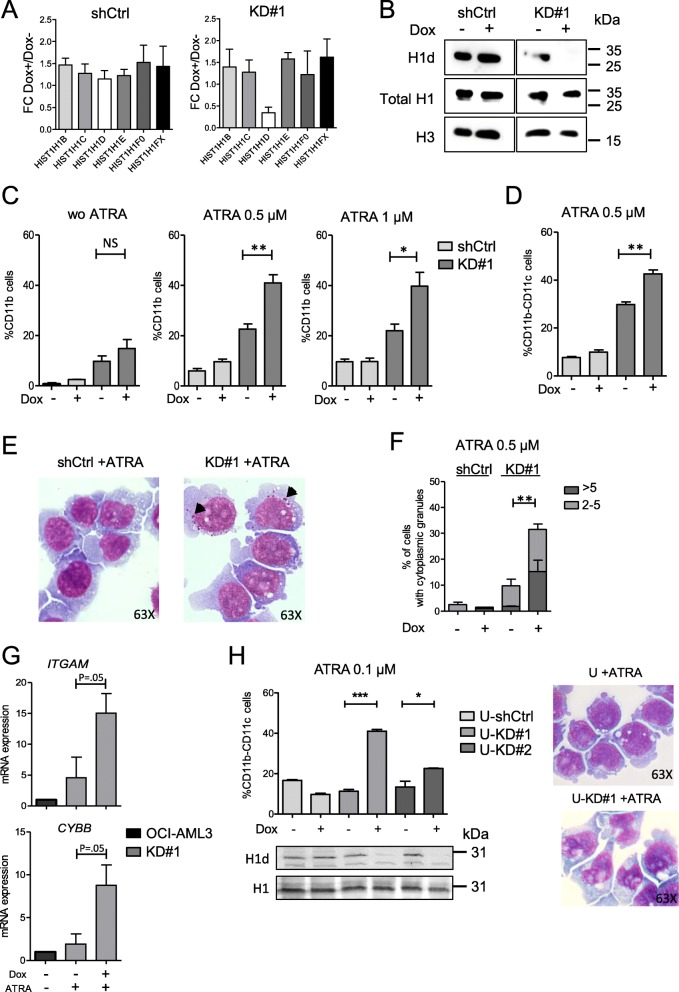
H1-3 KD promotes granulocytic differentiation in ATRA-treated AML cell lines. **a** qPCR expression analysis of the indicated histone H1 genes in shCtrl and shH1-3 (KD#1) without (Dox-) or with 6-days-induction of doxycycline (Dox+). Data represent three independent Dox inductions. Results are normalized on *HPRT* and expressed in fold change (FC) between Dox+ and Dox− conditions. **b** Immunoblot of H1-3, H1, and H3 in chromatin-bound extracts from shCtrl and KD#1 upon (+) or not (−) doxycycline (Dox). **c**, **d** Cell surface analysis of granulocytic markers consecutive to H1-3 KD in OCI-AML3 clones. **c** Percentage of CD11b positive cells in shCtrl and KD#1 upon doxycycline induction (Dox+) or not (Dox−) without ATRA (wo ATRA) or with 72 h of ATRA-treatment (0.5 μM or 1 μM). **d** Percentage of CD11b-CD11c cells upon 72 h of ATRA-treatment (0.5 μM). Data represent three independent experiments. Statistical significance was estimated using Mann Whitney test *p < 0.05; **p < 0.005. *NS* non-significant. **e**, **f** Cytological analysis of shCtrl and KD#1 clones upon doxycycline and after 96 h of ATRA treatment (0.5 μM). **e** May–Grünwald Giemsa coloration. Black arrows are pointing cytoplasmic azurophilic granules. **f** Quantification of cells with cytoplasmic granules. Results are presented as a percentage of positive cells (more than two granules). Statistical significance was estimated using Mann–Whitney test *p < 0.05; **p < 0.005. **g** Expression analysis of *CYBB* and *ITGAM* in untreated OCI-AML3 and in ATRA-treated (1 μM) KD#1 without or with doxycycline induction. Gene expression was normalized to two housekeeping genes (*PGK1* and *PPIA*). Data represent three independent experiments. Statistical significance was estimated using T-test (one-tailed *p* value). **h** U937 cells were stably infected by a doxycycline inducible shCtrl (U-shCtrl) or shH1-3 (U-KD#1 and U-KD#2). Left panel: percentage of CD11b-CD11c cells upon 72 h of ATRA-treatment (0.1 μM). Level of H1d in the different conditions was measured by immunoblot. Right panel: May–Grünwald Giemsa staining of KD#1 clone with or without doxycycline upon 72 h hours of ATRA-treatment. Data represent two independent experiments. Statistical significance was estimated using unpaired Two-tailed *t* test * *p* < 0.05; ***p < 0.001

We hypothesized that the better prognosis of H1-3 low AML may be related to a gain of differentiation and a loss of stem cell features of the AML blasts. Thus, we tested the consequences of H1-3 knockdown (KD) on differentiation potential of the *NPM1*-mutated OCI-AML3 cell line, which also harbor the *DNMT3A*R882C mutation [21]. Efficiency and specificity of our KD were assessed by testing mRNA levels of the different H1 subtypes (Fig. 6a and Additional file 2: Figure S6a and S6b) and by measuring H1-3 protein level, after H1-3 KD induction (Fig. 6b and Additional file 2: Figure S6c and S6d). Consequences of H1-3 KD on differentiation were evaluated upon all-trans retinoic acid (ATRA)-treatment. Albeit no increase in CD11b was observed upon H1-3 KD alone, addition of ATRA (0.5 μM and 1 μM) induced a significant increase in CD11b expression, with a marked increase at 0.5 μM (22.6 ± 2.5% vs. 41 ± 4.3%; *P* = .008) (Fig. 6c and Additional file 2: Figure S7a and S7b) and a significant increase in the proportion of the double positive CD11b/CD11c population (29.8 ± 1.3% vs. 42.5 ± 2.1%; *P* = .003) (Fig. 6d). In addition, morphological and quantification analyses showed that cytoplasmic granules, which reflect the beginning of a maturation process appeared upon H1-3 KD after 96 h of ATRA-treatment (0.5 μM), (Fig. 6e, f and Additional file 2: Figure S8). Finally, mRNA expression levels of two ATRA-induced genes, *CYBB* and *ITGAM*, were tested in H1-3 KD condition under ATRA-treatment; H1-3 downregulation increased the amplitude of ATRA-induced upregulation of these two genes (Fig. 6g). To test whether this ATRA-sensitization was dependent of the presence of *NPM1*mut or *DNMT3A*R882C, we performed H1-3 KD in the *NPM1*wt and *DNMT3A*wt myeloid U937 cell line. Interestingly, H1-3 KD increased the proportion of the double positive CD11b/CD11c in 0.1 μM ATRA-treated U937 cells and induced morphological changes (Fig. 6h). Altogether, these results suggest that downregulation of histone H1-3 induces ATRA-sensitization independently of *NPM1* and *DNMT3A* mutations.

## Discussion

Aberrant epigenetic patterns in leukemia have been described but determination of their importance in leukemia onset and progression is still lacking [22]. We have studied here the clinical and biological consequences of an epigenetic alteration of the *HIST1* locus, the H3K27me3 *HIST1* signature, which we previously described in CN-AML [10]. We first confirmed the presence of H3K27me3 *HIST1*^high^ on 55% of CN-AML and 75% of *NPM1*mut CN-AML in an independent CN-AML cohort. H3K27me3 *HIST1*^high^ impacts on the survival of CN-AML and *NPM1*mut CN-AML patients, and confers a better survival independently of other molecular markers. While H3K27me3 *HIST1*^high^ is not associated with *FLT3*ITD or *DNMT3A* mutations, *IDH2*R140Q is overrepresented in H3K27me3 *HIST1*^high^ patients (28% vs 7.6%); this is consistent with data highlighting the functional relationship between *IDH2*R140Q mutant expression and histone hypermethylation [23]. Although we do not know the mechanisms underlying the H3K27me3 *HIST1*^high^ signature, this focal high level of H3K27me3 may reflect a maintained activity of EZH2 previously shown to be associated with favorable outcome in AML [24]. Our data provide the first evidence that detecting an aberrant epigenetic pattern in addition to gene mutations has clinical interest and could guide therapeutic choices.

Detection of our epigenetic biomarker in AML demonstrates that H3K27me3 *HIST1*^high^ status is invariably associated with a favorable outcome and may reveal a less aggressive disease. The less aggressive form of AML associated with H3K27me3 *HIST1*^high^ is supported by its anti-correlated stem cell signature, shown to be associated with an adverse prognosis [19]. It is also supported by its inflammatory and immune function signature, which has been associated with a better clinical response to dexamethasone in NPM1 CN-AML [25]. However, although CD34low AMLs, characterized by a leukemic stem cell arrested at a precursor-like stage [26], are enriched in the H3K27me3 *HIST1*^high^ group, we could not find H3K27me3 *HIST1*^high^ exclusively on CD34low blasts. This suggests that H3K27me3 *HIST1*^high^ marks a more mature leukemia independently of the level of CD34 expression.

The main consequence of the H3K27me3 accumulation at the *HIST1* locus may be the downregulation of the histone genes affected by the repressive epigenetic mark. Levels of replication-dependent histone gene expression may reflect aggressiveness of the disease and may have a survival impact. Indeed, the major histone gene cluster has been described as one of the most upregulated across breast cancer progression [9]. Our study highlighted a peculiar pattern of a lower histone gene expression and defined a three-*HIST1* mRNA signature, containing three histone genes (*HIST1H1D*, *HIST1H2BH*, and *HIST1H2BG*) that are directly affected by the H3K27me3 enrichment and can predict survival on CN-AML patients. Interestingly, one of the most affected histone genes in our AML data encodes the histone linker H1-3*.* High level of H1.3 has been previously linked to cancer aggressiveness, as its overexpression is associated with malignant ovarian adenocarcinoma [27] and with a poor pancreatic ductal adenocarcinoma survival [28]. Given its tight link with differentiation blockage in cancer cells [29] and given that H1 subtype individual KDs induce changes, notably in genes involved in cell cycle and chromatin regulation [30], diminution of H1.3 level appears to be a valid effector of the less aggressive H3K27me3 *HIST1*^high^ AML phenotype. If histone linker H1 subtypes have a well-described redundant roles in maintaining nucleosome architecture and regulating transcription [31], it becomes evident that H1 subtypes have also subtype-specific functions reflected by selective genomic binding and regulation of chromatin organization [20, 32]. H1 subtypes would influence chromatin compaction at definite loci affecting specific gene expression in a tissue specific manner. Thus, it is tempting to speculate that in our H3K27me3 *HIST1*^high^ model, low H1-3 level would influence the differentiation state of the cells due to a specific change in chromatin targeting. In our cellular models, KD of H1-3 does not induce differentiation but sensitizes the cells to ATRA-treatment. Interestingly, this sensitivity is independent of DNMT3A activity, as the two cell line models (OCI-AML3 *DNMT3A*mut and U937 *DNMT3A*wt) are both sensitive to ATRA-treatment after H1-3 KD. In the light of previous reports, which documented an interaction between H1 proteins and DNMT3B [33, 34], one possibility is that *DNMT3B*, although not found mutated in AML, may be an influential epigenetic partner in AML and in ATRA sensitivity together with H1-3.

ATRA is successfully used for the treatment of acute promyelocytic leukemia (APL) by inducing terminal granulocytic differentiation of APL blasts [35]. To a lesser extent, ATRA can also induce differentiation in non-APL cells, in particular in AML with *IDH1*/*IDH2* mutations [36] or induce cell death in *NPM1*mut AML when used in combination with Arsenic trioxide [37], [38]. Interestingly, ATRA sensitization of non-APL cells has been induced by targeting epigenetic enzymes: inhibition of histone deacetylase (HDAC) with valproic acid [39] or inhibition of the histone demethylase LSD1/KDM1A [40] or by targeting SUMOylation [41]. In the same line, our study suggests that epigenetic modifications induced by H1-3 targeting could prime AML cells toward differentiation, revealed by ATRA-sensitivity, which may explains the more mature phenotype found in H3K27me3 *HIST1*^high^ leukemia and suggests that ATRA could be an efficient differentiating agent in AML with low H1-3 expression.

## Conclusions

We showed that epigenetic silencing of a part of the *HIST1* locus by the H3K27me3 mark is associated with a better outcome and a mature gene expression profile in *NPM1*mut CN-AML and we observed an important role of histone linker H1-3 expression in AML blast cell differentiation. Our study pinpoints the H3K27me3 *HIST1* mark and *HIST1H1D* gene as two biomarkers potentially useful to stratify patient prognosis and defines targets that can be considered when developing epidrugs.

## Methods

### Patient samples

Blast cells were separated from blood or marrow samples through density-gradient (ficoll) separation, and stored in liquid nitrogen. Cryopreserved samples, with at least 70% of blasts, were collected from AML samples stored at Institut Paoli-Calmettes (IPC) Tumor Bank or at the Groupe Ouest Est d’Etude des Leucémies Aiguës et autres Maladies du Sang repository (GOELAMSTHEQUE). GOELAMS samples were extracted from multicenter clinical trials LAM2006IR (NCT00860639) or LAM2007SA (NCT00590837). All patients received conventional induction chemotherapy consisting in daunorubicine (DNR) and aracytine (ARAC) with or without Mylotarg in the LAM2006IR trial for patients < 60 years [42] and idarubicine (IDA) and ARAC with or without lomustine in the LAM2007SA trial for patients ≥ 60 years [43]. Informed consent was provided by all patients according to the Declaration of Helsinki and subjected to ethical institutional review board approval.

### ChIP-qPCR

Chromatin immunoprecipitation (ChIP) was performed as previously described [10]. Briefly, frozen samples were thawed, washed twice in phosphate-buffered saline (PBS), and chromatin was extracted with TRIS buffer pH 8, 0.25% Triton. Samples were sonicated to obtain DNA fragments of 300–600 base pair (Bioruptor PICO) and chromatin was immunoprecipated with an anti-H3K27me3 antibody (Abcam #6002). After immunoprecipitation, DNA was purified with the I-Pure kit (Diagenode). Quantification of ChIPed DNA was performed by real-time PCR using the SsoADV Univer SYBR Green Supermix (Bio-Rad) and detected with a CFX96 Real-Time PCR Detection System (Bio-Rad). IgG control “cycle over the threshold” Ct values were subtracted to input or IP Ct values and converted into bound value by 2^(−(IP Ct or input Ct- IgG IP Ct))^. Data are expressed as percent of bound/input. For each *HIST1* region, H3K27me3 ChIP signal was double normalized with ChIP signal obtained at a genomic location invariably enriched with H3K27me3 (*HOXD4*) and a genomic region depleted of H3K27me3 (*GAPDH*). Heatmaps were performed with gene-e software (Broad institute). Hierarchical clustering was done using Euclidian distance.

### Histone gene nomenclature

The large *HIST1* gene cluster on human chromosome region 6p22 is 2.1 Mb which contains 55 histone genes. Five genes (*HIST1H1A-E*) encode the canonical somatic histone linkers H1 (H1-1, H1-5, H1-2, H1-3, H1-4 respectively) while sets of 10–20 genes encode each of the core histone proteins (H2A, H2B, H3, and H4). Each of these genes is translated into a unique mRNA with distinct 5′ and 3′ extremities, as well as slight nucleotide changes in the coding region.

### RT-qPCR analysis

Total RNA was isolated from patient samples using the RNeasy mini Kit (Qiagen). RNA was treated with RNase-free DNase set (Qiagen) to remove contaminating genomic DNA. The cDNA was synthesized using the Transcriptor High Fidelity cDNA Synthesis Kit (Roche Applied Science), quantified by Power SYBR Green (Roche Applied Science) or TaqMan qPCR. For SYBR green analyses, signals were detected with a CFX96 Real-Time PCR Detection System (Bio-Rad). Primers used for gene amplification were designed using Primer3 software and are listed in the table below. Relative expression levels were determined by the delta Ct method and expression level of *HPRT* was used for normalization. For TaqMan method, PCR Master Mix (Thermo Fisher) was used and signal detected with 7500 Fast Real-Time PCR System (Applied Biosystem). Probes used for TaqMan analyses were *HIST1H1D*: Hs00271187_s1; *HIST1H2BG*: Hs00374317_s1; *HIST1H2BH*: Hs00374322_s1; *PGK1*: Hs00943178_g1; *PPIA:* Hs04194521_s1 (Thermo Fisher). Relative expression levels were determined by the delta Ct method, taking the mean expression level of *PKG1* and *PPIA* for normalization [44].

### Gene expression profiling

RNA expression profiling of *NPM1*mut CN-AML was done with Affymetrix Human gene ST 2.0 and Human Genome U133 Plus 2.0 DNA microarrays (see Supplemental data). Microarray data are accessible under the accession number E-MTAB-6997.

### Protein analysis

Cellular fractionation was done with the subcellular protein fractionation kit (Thermofisher). Mass spectrometry procedures are explained in supplemental data. Immunoblots were done as previously described [45]. Antibodies used were anti-Histone H1.3 (Abcam, ab24174, 1/750), anti-H1 (Active Motif, #39707, 1/2000), and anti-H3 (Active Motif, #39163, 1/10000).

### Flow cytometry

Flow cytometry analyses were done using a BD-LSRII cytometer and analyzed using BD-DIVA Version 6.1.2 software (BD Biosciences). Antibodies used were CD11B-PE (Mac-1), 3:100, Beckman Coulter; CD11B-APC (M1/70), 1:500, eBioscience; CD11C-PeCy7 (BU-15), 3:100, Beckman Coulter; DRAQ7™, 1:400; Biostatus, CD34-PeCy7 (#343516) 1:33, Biolegend.

### Cell culture, shRNA lentiviral infection, stable H1-3 knockdown, and treatments

The OCI-AML3 and U937 cells were grown in minimum essential medium alpha (MEMα) supplemented with 20% fetal bovine serum or Roswell Park Memorial Institute (RPMI) medium supplemented with 10% fetal bovine serum respectively. H1-3 knockdown (KD) was achieved using doxycycline-induced Dharmacon™SMARTvector™ short hairpin RNA (V3SH7669-229784413, shRNA-1 and V3SH7669-228676834, shRNA-2). A non-silencing shRNA (piSMART VSC10730) was used as a control (shCtrl). Cells containing the SMARTvector™ were sorted using an ARIAIII cytometer based on red fluorescent protein (RFP) expression and selected on puromycin (2 μg/mL). KD of H1-3 was obtained by the addition of doxycycline (2 μg/mL) during 5–7 days. All-*trans*-retinoic acid (ATRA; Sigma) was dissolved in dimethylsulfoxyde (DMSO) at 10 mM.

### Statistical analyses

Statistical analyses were done using R software (version 2.15.2) (The Comprehensive R Archive Network. http://www.cran.r-project.org/) and Graph Pad Prism (Graph Pad Software, San Diego, CA, USA) and the significance of the differences between groups was determined via unpaired *T* test, Mann–Whitney test, or exact Fisher test. Data were presented as the median ± SEM. Overall survival (OS) and event-free survival (EFS) were calculated from the date of diagnosis to the date of death or to the date of relapse, death or the time to no response to intensive induction, respectively. Follow-up was measured from the date of diagnosis to the date of last news for living patients. Survivals were calculated using the Kaplan–Meier method and were compared with the log-rank test. Uni- and multivariate survival analyses were done using Cox regression analysis (Wald test). Variables with a *p* value < 0.05 were tested in multivariate analysis. All statistical tests were two-sided at the 5% level of significance, except when indicated otherwise.

## Supplementary information

Additional file 1. Table S1. Clinical and molecular characteristics in the GOELAMS Cohort (*n* = 46) according to H3K27me3 *HIST1* status. Table S2. Multivariate analyses in the validation CN-AML cohort (n = 46). Table S3. Univariate and Multivariate Analyses for 3-*HIST1*-mRNA signature in TCGA and Metzeler cohorts. Table S4. Clinical characteristics of *NPM1*mut patients selected for transcriptomic analysis.

Additional file 2. Figure S1. Related to Fig. 2: Heatmap of the H3K27me3 level in a new cohort of 78 *NPM*1mut CN-AML patients. Figure S2. Related to Fig. 2: Analysis of H3K27me3 *HIST1* status in CD34low and CD34high sorted blasts. Figure S3 Related to Fig. 4. Representative Integrative Genomics Viewer (IGV) tracks of H3K27me3 signal obtained from ChIP-chip data published in Tiberi *et al*., 2015 Figure S4. Related to Fig. 5: Histone protein extraction in *NPM1*mut patients. Figure S5. Related to Fig. 5:Total protein abundance of each histone types determined by IBAQ label-free quantification method. Figure S6 related to Fig. 6. Effect of H1-3 KD on histone H1 subtype mRNA and protein expression. Figure S7 related to Fig. 6. Effect of H1d KD on CD11b expression in shRNA1 (clones KD#2 and KD#3) and in shRNA2 conditions. Figure S7 related to Figure 6. Effect of H1d KD on CD11b expression in shRNA1 (clones KD#2 and KD#3) and in shRNA2 conditions

Additional file 3. Supplemental Methods

Additional file 4. Table S5. GES 81 g, LAM HIST1 16 ‘high (1)’ vs. 11 ‘low (0)’

## Abbreviations

APL: Acute promyelocytic leukemia
ARAC: Aracytine
ATRA: All-trans retinoic acid
CEBPA: CCAAT/enhancer-binding protein alpha
ChIP: Chromatin immunoprecipitation
CN-AML: Cytogenetically normal acute myeloid leukemia
DMSO: Dimethylsulfoxyde
DNMT3A: DNA-methyl transferase 3A
DNR: Daunorubicine
EFS: Event-free survival
EZH2: Enhancer of zeste homolog 2
FACS: Fluorescence Activated Cell Sorting
FLT3: Fms-like tyrosine kinase 3
H3K27me3: Histone H3 Lysine 27 tri-methylation
HIST1: Histone cluster 1
HR: Hazard ratio
IDA: Idarubicine
IDH: Isocitrate dehydrogenase
ITD: Internal tandem duplications
KD: Knock down
KDM1/LSD1: Lysine specific demethylase 1
MEMα: Minimum essential medium alpha
NPM1: Nucleophosmin 1
OS: Overall survival
PBS: Phosphate-buffered saline
RFP: Red fluorescent protein
RPMI: Roswell Park Memorial Institute medium
SUZ12: Suppressor of zeste 12
