# Supplementary material for: Epigenetic down-regulation of the HIST1 locus predicts better prognosis in acute myeloid leukemia with NPM1 mutation
Source: Clin Epigenetics. 2019 Oct 12;11:141. doi: 10.1186/s13148-019-0738-6 (PMC6790061; doi:10.1186/s13148-019-0738-6)
Supplement: Supplementary file 1 — Table S1. Clinical and molecular characteristics in the GOELAMS Cohort (n = 46) according to H3K27me3 HIST1 status. Table S2. Multivariate analyses in the validation CN-AML cohort (n = 46). Table S3. Univariate and Multivariate Analyses for 3-HIST1-mRNA signature in TCGA and Metzeler cohorts. Table S4. Clinical characteristics of NPM1mut patients selected for transcriptomic analysis. [file 13148_2019_738_MOESM1_ESM.docx]

**Supplemental Tables**

**Table S1: Clinical and molecular characteristics in the GOELAMS Cohort (n=46) according to H3K27me3 *HIST1* status**

| **Characteristics** | | **All patients (n=46)** | **H3K27me3 *HIST1*^low^ (n=21)** | **H3K27me3 *HIST1*^high^ (n=25)** | ***P*** |
| --- | --- | --- | --- | --- | --- |
| Age, y | |  |  |  | 0,37 |
|  | Median | 62.8 | 63.2 | 62.4 |  |
|  | Range | 28-76 | 28-76 | 43-75 |  |
| Sex, % | |  |  |  | 1.0 |
|  | Male | 52.1 | 52.4 | 52 |  |
| Complete response, % | | 80.4 | 71.4 | 88.0 | 0.26 |
| Molecular genetics, % | |  |  |  |  |
|  | *NPM1* | 71.7 | 38.1 | 100 | <0.001 |
|  | *FLT3*ITD | 47.8 | 47.6 | 48.0 | 1 |
|  | *DNMT3A* | 19.6 | 23.8 | 16.0 | 0.71 |
|  | *IDH2* (R140) | 21.7 | 4.8 | 36.0 | 0.01 |
|  | *IDH1* (R132) | 15.2 | 9.5 | 20.0 | 0.22 |
|  | *CEBPA* | 15.2 | 23.8 | 8.0 | 0.46 |
|  | *ASXL1* | 2.1 | 4.7 | 0.0 | 0.46 |

**Table S2: Multivariate analyses in the validation CN-AML cohort (n=46)**

| Variables | HR | 95% CI | *P* |
| --- | --- | --- | --- |
| **Event free survival** |  |  |  |
| H3K27me3 *HIST1*^high^ | 3.15 | 1.36-7.33 | 0.008 |
| Age > 60 years | 0.34 | 0.16-0.77 | 0.01 |
| *NPM1*wt/*FLT3*ITD | 0.99 | 0.35-2.84 | 0.99 |
| **Overall survival** |  |  |  |
| H3K27me3 *HIST1*^high^ | 2.34 | 0.98-5.62 | 0.06 |
| Age > 60 years | 0.38 | 0.16-0.89 | 0.03 |
| *NPM1*wt/*FLT3*ITD | 0.54 | 0.19-1.52 | 0.24 |

**Table S3: Univariate and Multivariate Analyses for 3-*HIST1*-mRNA signature in TCGA and Metzeler cohorts**

| **Overall free survival** | **Univariate** | | | | **Multivariate** | | | |
| --- | --- | --- | --- | --- | --- | --- | --- | --- |
| Variables | N | HR | 95% CI | *P* | N | HR | 95% CI | *P* |
| age | 188 | 1.02 | 1.00-1.03 | 0.008 | 188 | 1.02 | 1.00-1.03 | 0.01 |
| gender (male vs. female) | 52 | 0.77 | 0.39-1.53 | 0.462 | - | - | - | - |
| FAB classification | 188 | 1.27 | 0.30-5.27 | 0.7 | - | - | - | - |
| *FLT3 (mut vs. wt)* | 188 | 1.71 | 1.18-2.48 | 0.004 | 188 | 1.65 | 1.14-2.39 | 0.008 |
| *3-HIST1 mRNA signature* | 188 | 1.73 | 1.20-2.50 | 0.003 | 188 | 1.6 | 1.11-2.31 | 0.01 |

**Table S4: Clinical characteristics of *NPM1*mut patients selected for transcriptomic analysis**

| **Variables** | **All patients (n=27)** | **H3K27me3 HIST1^low^ (n=11)** | **H3K27me3 HIST1^high^ (n=16)** |
| --- | --- | --- | --- |
| Median age | 60 | 63 | 53 |
| Male sexe, n | 12 | 5 | 7 |
| FAB 1-2 *vs* 4-5, n | 13 *vs* 14 | 6 *vs* 5 | 7 *vs* 9 |
| *FLT3*ITD, n | 12 | 5 | 7 |
