# Supplementary material for: Epigenetic down-regulation of the HIST1 locus predicts better prognosis in acute myeloid leukemia with NPM1 mutation
Source: Clin Epigenetics. 2019 Oct 12;11:141. doi: 10.1186/s13148-019-0738-6 (PMC6790061; doi:10.1186/s13148-019-0738-6)
Supplement: Supplementary file 2 — Figure S1. Related to Fig. 2: Heatmap of the H3K27me3 level in a new cohort of 78 NPM1mut CN-AML patients. Figure S2. Related to Fig. 2: Analysis of H3K27me3 HIST1 status in CD34low and CD34high sorted blasts. Figure S3 Related to Fig. 4. Representative Integrative Genomics Viewer (IGV) tracks of H3K27me3 signal obtained from ChIP-chip data published in Tiberi et al., 2015 Figure S4. Related to Fig. 5: Histone protein extraction in NPM1mut patients. Figure S5. Related to Fig. 5:Total protein abundance of each histone types determined by IBAQ label-free quantification method. Figure S6 related to Fig. 6. Effect of H1-3 KD on histone H1 subtype mRNA and protein expression. Figure S7 related to Fig. 6. Effect of H1d KD on CD11b expression in shRNA1 (clones KD#2 and KD#3) and in shRNA2 conditions. Figure S7 related to Figure 6. Effect of H1d KD on CD11b expression in shRNA1 (clones KD#2 and KD#3) and in shRNA2 conditions [file 13148_2019_738_MOESM2_ESM.pptx]

## Slide 1
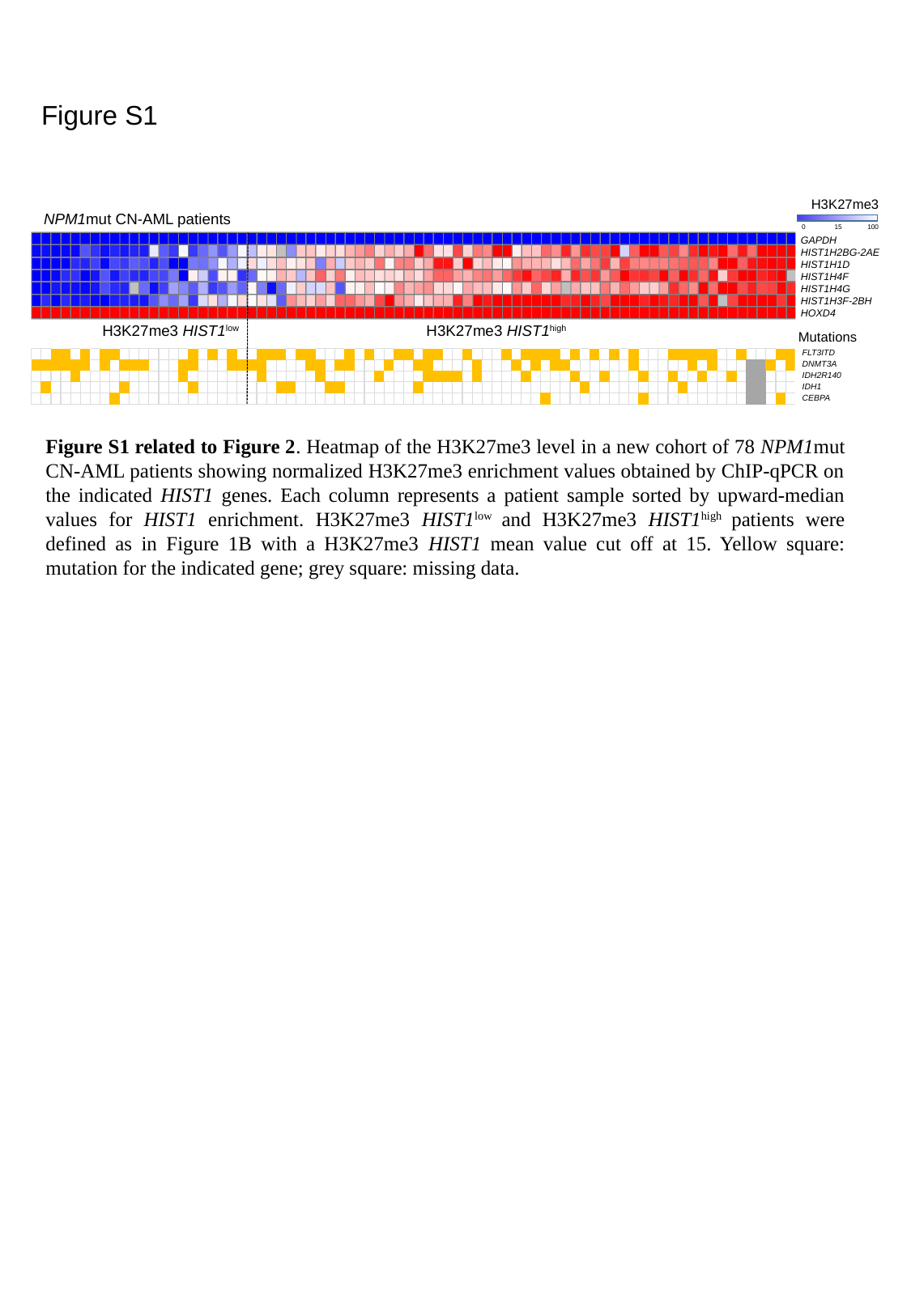

Figure S1
H3K27me3
15
0
100
NPM1mut CN-AML patients
GAPDH
HIST1H2BG-2AE
HIST1H1D
HIST1H4F
HIST1H4G
HIST1H3F-2BH
HOXD4
H3K27me3 HIST1low
H3K27me3 HIST1high
Mutations
| FLT3ITD |
| --- |
| DNMT3A |
| IDH2R140 |
| IDH1 |
| CEBPA |
Figure S1 related to Figure 2. Heatmap of the H3K27me3 level in a new cohort of 78 NPM1mut CN-AML patients showing normalized H3K27me3 enrichment values obtained by ChIP-qPCR on the indicated HIST1 genes. Each column represents a patient sample sorted by upward-median values for HIST1 enrichment. H3K27me3 HIST1low and H3K27me3 HIST1high patients were defined as in Figure 1B with a H3K27me3 HIST1 mean value cut off at 15. Yellow square: mutation for the indicated gene; grey square: missing data.

## Slide 2
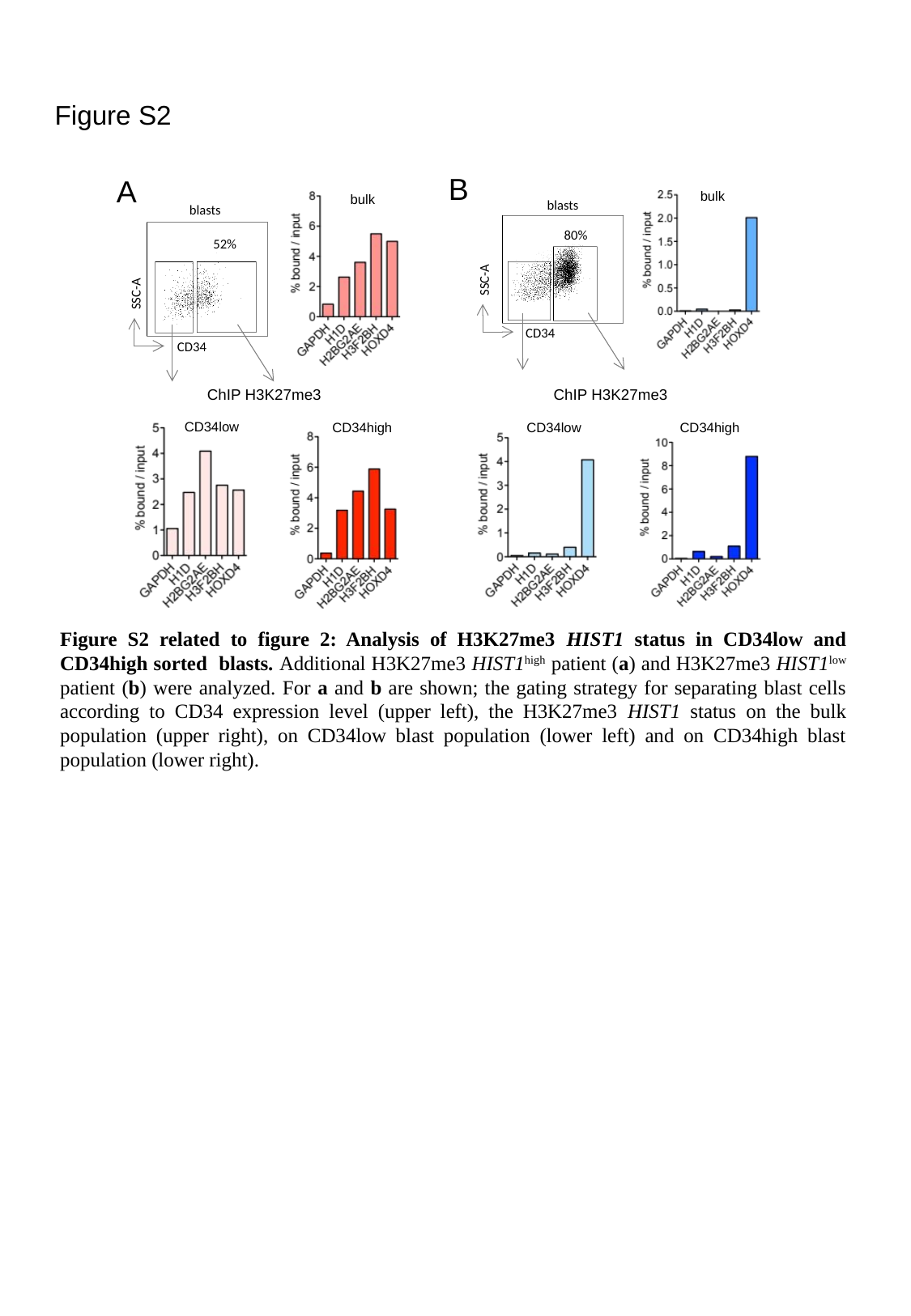

Figure S2
B
A
bulk
bulk
blasts
80%
SSC-A
CD34
blasts
52%
SSC-A
CD34
ChIP H3K27me3
ChIP H3K27me3
CD34low
CD34high
CD34high
CD34low
Figure S2 related to figure 2: Analysis of H3K27me3 HIST1 status in CD34low and CD34high sorted blasts. Additional H3K27me3 HIST1high patient (a) and H3K27me3 HIST1low patient (b) were analyzed. For a and b are shown; the gating strategy for separating blast cells according to CD34 expression level (upper left), the H3K27me3 HIST1 status on the bulk population (upper right), on CD34low blast population (lower left) and on CD34high blast population (lower right).

## Slide 3
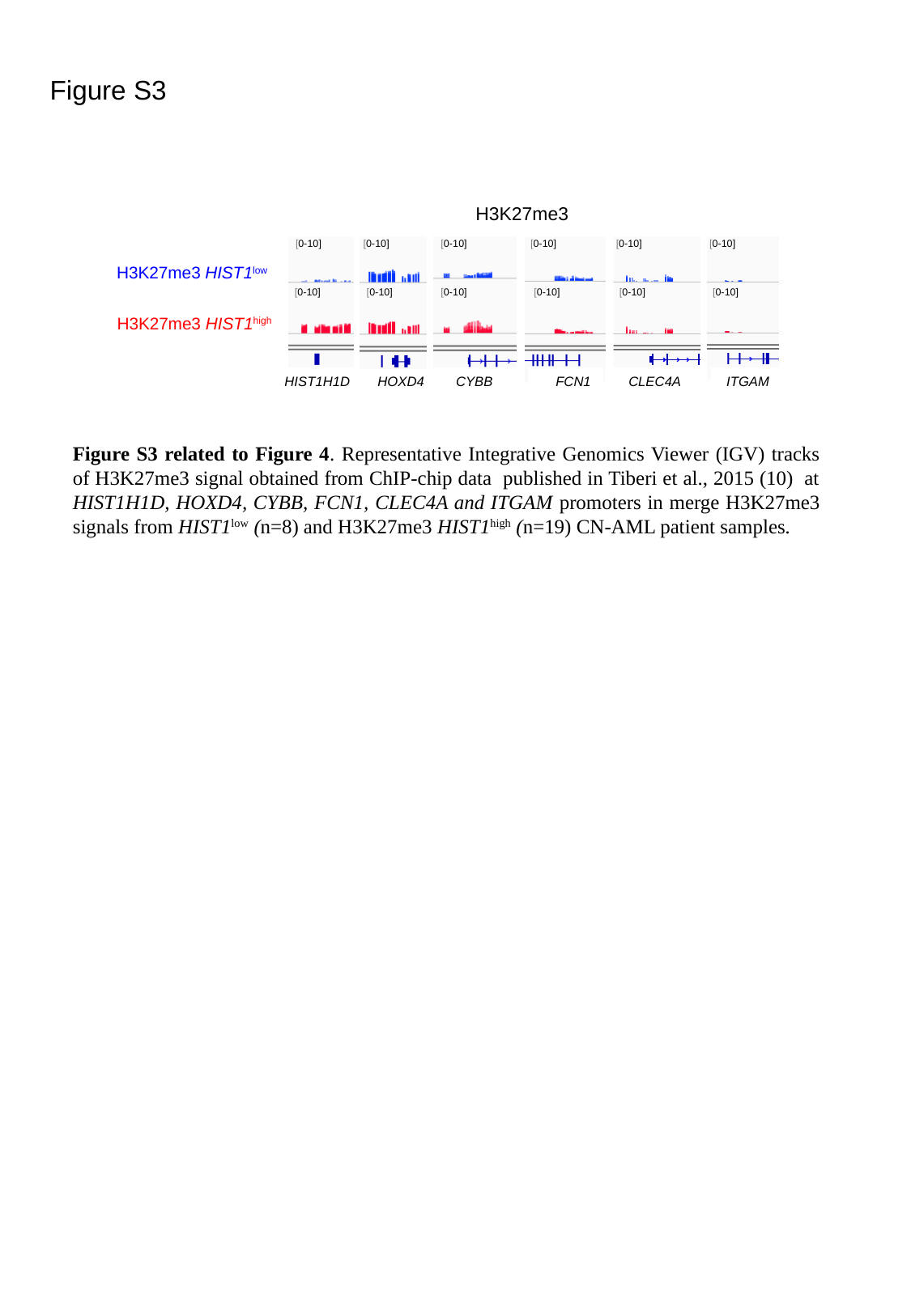

Figure S3
H3K27me3
[0-10]
[0-10]
[0-10]
[0-10]
[0-10]
[0-10]
[0-10]
[0-10]
[0-10]
[0-10]
[0-10]
[0-10]
H3K27me3 HIST1low
H3K27me3 HIST1high
HIST1H1D
HOXD4
 CYBB
 FCN1
 CLEC4A
 ITGAM
Figure S3 related to Figure 4. Representative Integrative Genomics Viewer (IGV) tracks of H3K27me3 signal obtained from ChIP-chip data published in Tiberi et al., 2015 (10) at HIST1H1D, HOXD4, CYBB, FCN1, CLEC4A and ITGAM promoters in merge H3K27me3 signals from HIST1low (n=8) and H3K27me3 HIST1high (n=19) CN-AML patient samples.

## Slide 4
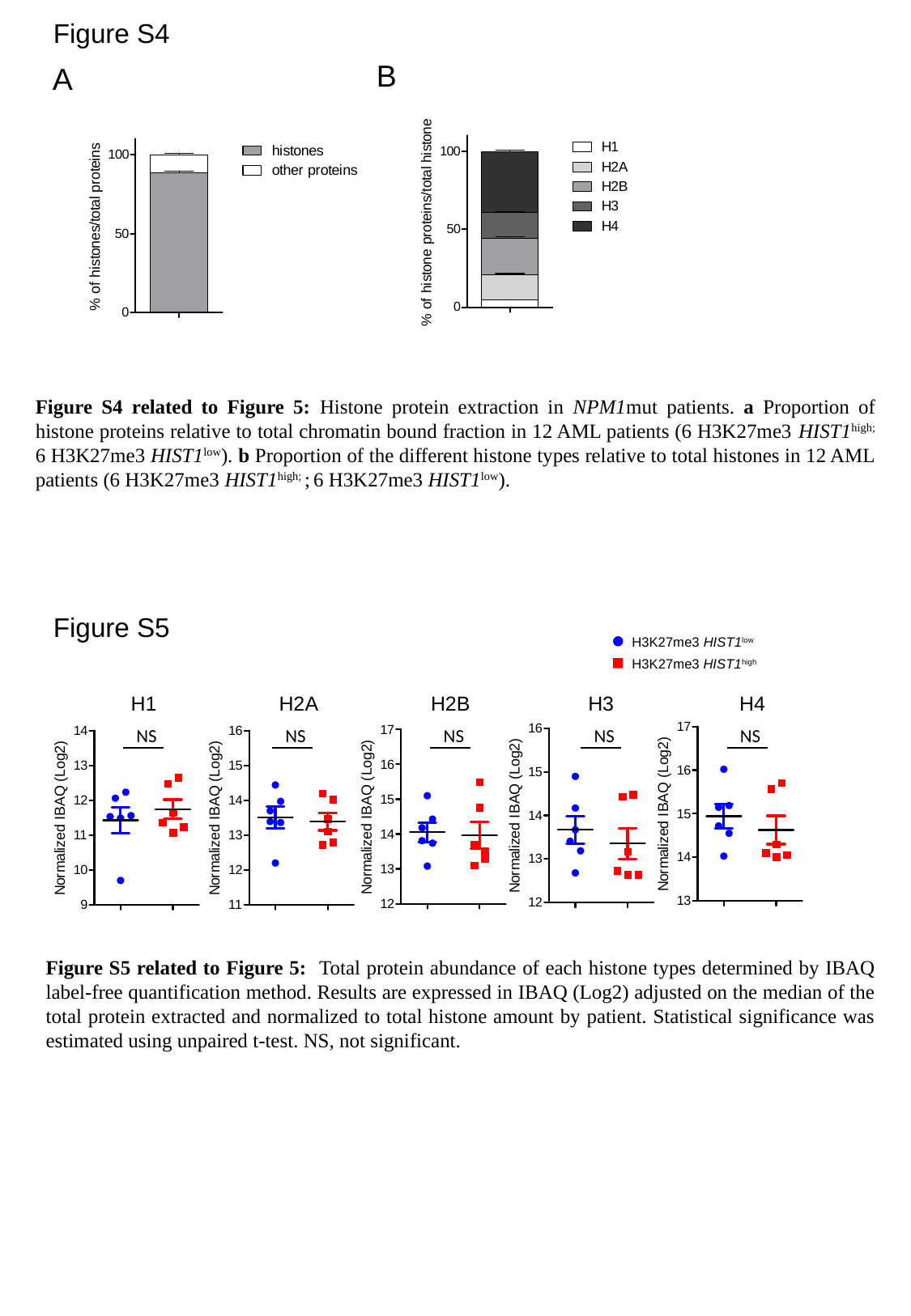

Figure S4
B
A
Figure S4 related to Figure 5: Histone protein extraction in NPM1mut patients. a Proportion of histone proteins relative to total chromatin bound fraction in 12 AML patients (6 H3K27me3 HIST1high; 6 H3K27me3 HIST1low). b Proportion of the different histone types relative to total histones in 12 AML patients (6 H3K27me3 HIST1high; ; 6 H3K27me3 HIST1low).
Figure S5
H3K27me3 HIST1low
 H3K27me3 HIST1high
H1
H2A
H2B
H3
H4
NS
NS
NS
NS
NS
Figure S5 related to Figure 5: Total protein abundance of each histone types determined by IBAQ label-free quantification method. Results are expressed in IBAQ (Log2) adjusted on the median of the total protein extracted and normalized to total histone amount by patient. Statistical significance was estimated using unpaired t-test. NS, not significant.

## Slide 5
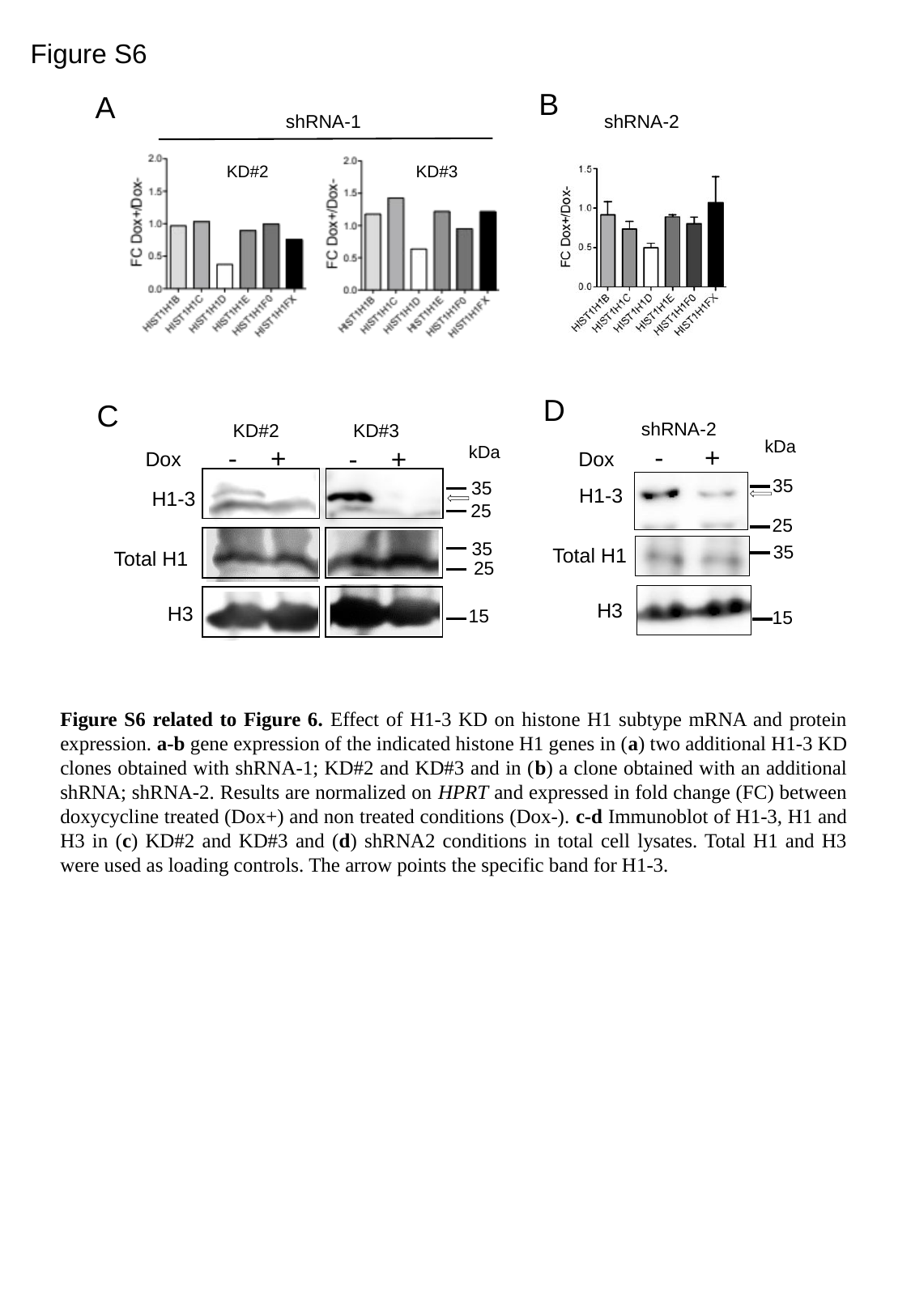

Figure S6
B
A
shRNA-1
shRNA-2
KD#2
KD#3
D
C
shRNA-2
KD#2
KD#3
-
+
-
+
Dox
35
H1-3
25
35
Total H1
25
H3
15
kDa
-
+
Dox
35
H1-3
25
35
Total H1
H3
15
kDa
Figure S6 related to Figure 6. Effect of H1-3 KD on histone H1 subtype mRNA and protein expression. a-b gene expression of the indicated histone H1 genes in (a) two additional H1-3 KD clones obtained with shRNA-1; KD#2 and KD#3 and in (b) a clone obtained with an additional shRNA; shRNA-2. Results are normalized on HPRT and expressed in fold change (FC) between doxycycline treated (Dox+) and non treated conditions (Dox-). c-d Immunoblot of H1-3, H1 and H3 in (c) KD#2 and KD#3 and (d) shRNA2 conditions in total cell lysates. Total H1 and H3 were used as loading controls. The arrow points the specific band for H1-3.

## Slide 6
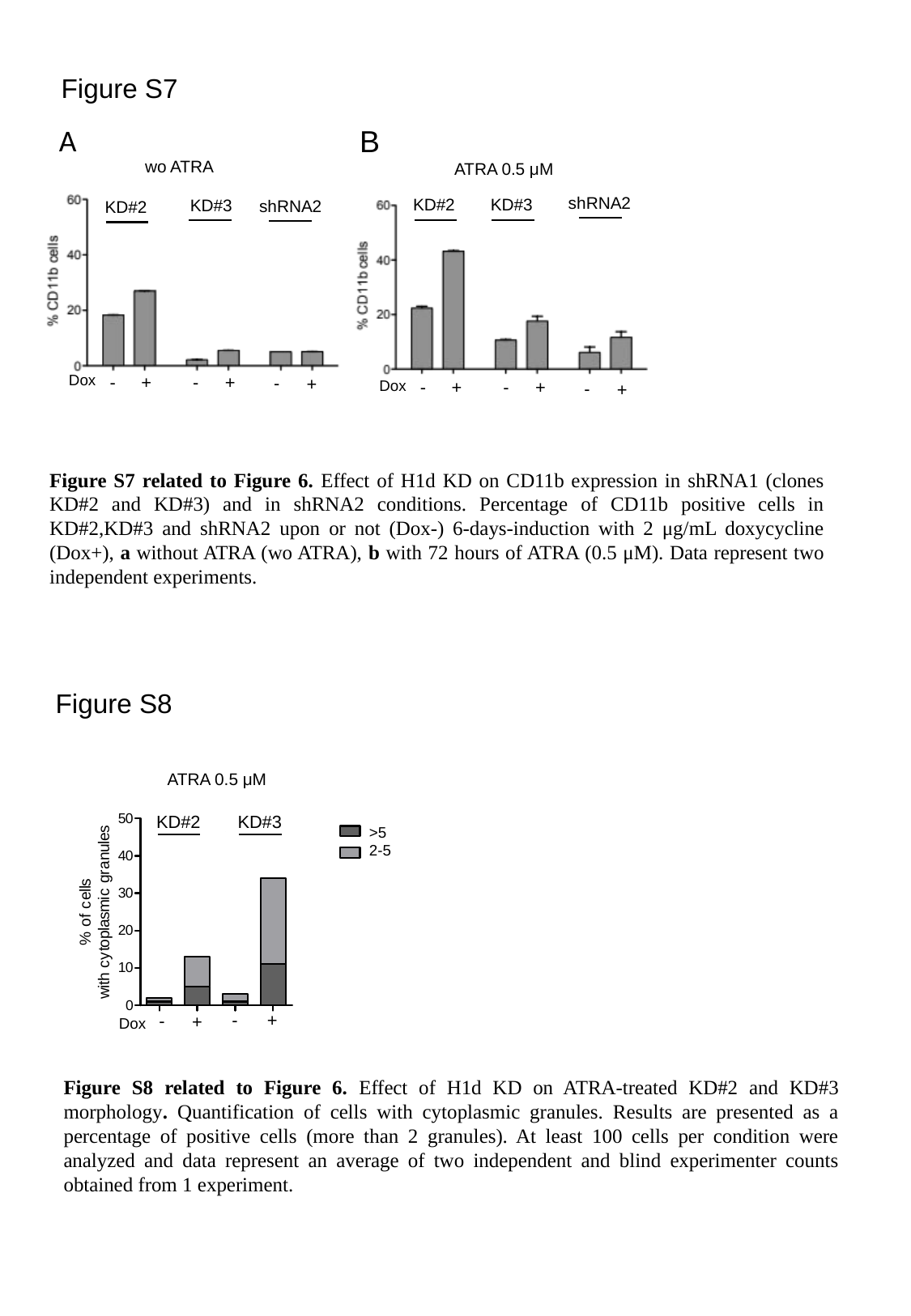

Figure S7
A
B
wo ATRA
ATRA 0.5 μM
shRNA2
KD#2
KD#3
KD#3
shRNA2
KD#2
Dox
-
+
-
+
-
+
Dox
-
+
-
+
-
+
Figure S7 related to Figure 6. Effect of H1d KD on CD11b expression in shRNA1 (clones KD#2 and KD#3) and in shRNA2 conditions. Percentage of CD11b positive cells in KD#2,KD#3 and shRNA2 upon or not (Dox-) 6-days-induction with 2 μg/mL doxycycline (Dox+), a without ATRA (wo ATRA), b with 72 hours of ATRA (0.5 μM). Data represent two independent experiments.
Figure S8
ATRA 0.5 μM
KD#2
KD#3
-
+
-
+
Dox
>5
2-5
Figure S8 related to Figure 6. Effect of H1d KD on ATRA-treated KD#2 and KD#3 morphology. Quantification of cells with cytoplasmic granules. Results are presented as a percentage of positive cells (more than 2 granules). At least 100 cells per condition were analyzed and data represent an average of two independent and blind experimenter counts obtained from 1 experiment.
