# Supplementary material for: Epigenetic down-regulation of the HIST1 locus predicts better prognosis in acute myeloid leukemia with NPM1 mutation
Source: Clin Epigenetics. 2019 Oct 12;11:141. doi: 10.1186/s13148-019-0738-6 (PMC6790061; doi:10.1186/s13148-019-0738-6)
Supplement: Supplementary file 3 — Supplemental Methods [file 13148_2019_738_MOESM3_ESM.docx]

**Supplemental Methods**

**Primers used for SYBR green qPCR**

| *Gene* | Forward primer | Reverse primer |
| --- | --- | --- |
| *HPRT* | GGCCCTCTGTGTGCTCAAG | CTGATAAAATCTACAGTCATAGGAATGGA |
| *HIST1H1B* | GTCAAAAAGGTGGCGAAGAG | CTTGGCCTTTGCAGCTTTAG |
| *HIST1H1C* | TGCCACTTGTACCCGAGTTT | CCTTTTTGGCCGCCTTCTTC |
| *HIST1H1D* | CTAAGGCCAAAGCCCCTAAG | CCGCCAGTTTCACTTTTTCT |
| *HIST1H1E* | GTCGGGTTCCTTCAAACTCA | GCCTTCTTTGGGGTCTTCTT |
| *HIST1H1F0* | CTCGCAGATCAAGTTGTCCA | GAAGGCCACTGACTTCTTGG |
| *HIST1H1FX* | GTGGTTCGACCAGCAGAATG | GAGCTTGAAGGAACCGTTGG |
| *HIST1H2AE* | AGTCAAATCCGTCAGTGATCC | TCAGGCATCGTATGTGTATTCC |
| *HIST1H2AI* | GCTACTGCCCAAGAAGACCGA | TTCCTTTGGGATTGAGTTGCTGC |
| *HIST1H2BG* | AAGGTACCAAGGCTGTCACC | TGTGAGACTTGAGTGGCTCTG |
| *HIST1H2BH* | CACTAAGGCCGTCACCAAGT | TTTGGGTTTGAACATGCGTC |
| *HIST1H3F* | CAAGCGAGTGACTATCATGCC | GCGCACACCCTCAGTACAAC |
| *HIST1H4F* | CCGTAACCTACACGGAGCAC | AAATAAAAAGCCGGGGTGAG |
| *CYBB* | AACTGGGCTGTGAATGAGGG | GCCAGTGCTGACCCAAGAA |
| *FCN1* | GATGCGTGGAGAGAAAGGAGAC | ATGTCACAGAGCACAGTCAGG |
| *CLEC4A* | GATCCAGAAGGTCAGCGACA | TGGATGCCAGAATGTGGAACT |
| *ITGAM* | CCCGGAAAACTCAGAGGTCAC | GCTGGTGACCACCATGTAGA |

**Gene expression profiling**

RNA expression profiling was done with Affymetrix Human gene ST 2.0 and Human Genome U133 Plus 2.0 DNA microarrays containing respectively more than 48,000 and 54,000 probe sets, representing 23,816 and 20,678 well-characterized human genes. Preparation of cRNA was done with the “GeneChip® WT PLUS Reagent Kit (Affymetrix)“ and “GeneChip® 3’ IVT Express Kit (Affymetrix)” respectively as recommended by the supplier. Hybridizations, washes, detection and quantification were performed as previously described (1). Expression data in both type of chips were normalized separately by the Robust Multichip Average method (2) using the Affymetrix “Expression Console” software. Next, we mapped hybridization probes based on their NCBI Entrez Gene identification number in both data sets. In the case of multiple probes by gene the one with the highest variance were selected. Finally, both data set were normalized and gathered using “inSilicoMerging” R-package with Empirical Bayes as method. To define specific genes of both H3K27me3 *HIST1* groups, a moderated t-Test analysis was applied to expression levels of each probe set using linear models with empirical Bayes statistic included in the limma R package (3). Probe sets were considered significantly as differentially expressed if they showed a fold change of at least 1.5 and a p-value of at least 5%. Analyses were done in R and associated packages. Gene set enrichment analysis (GSEA)(4) were made using Broad Institute tool (<http://www.broadinstitute.org/gsea/>). For GSEA analyses, gene selection was based on a p-value of at least 5% and a q-value of at least 30%.

**Gene expression data sets**

We collected two retrospective public gene expression data sets including CN AML samples profiled using Affymetrix oligonucleotide microarrays. Gene expression, molecular and clinical data were obtained from NCBI/GEO database and The Cancer Genome Atlas (TCGA) portal. We analyzed 329 AMLs with normal karyotype.

List of AML data sets included:

| **Source of data**  **(ref)** | **Technological platform** | **N° of probe sets/genes** | **Total (N)** | **AML (N)** | **CN-AML (N)** |
| --- | --- | --- | --- | --- | --- |
| TCGA portal (5) <https://tcga-data.nci.nih.gov> | Affymetrix,array U133 Plus 2.0 | 54K | 183 | 183 | 89 |
| GEO database, GSE12417 (6) | Affymetrix,array U133 A+B | 22K+22K | 242 | 240 | 240 |

**Pre-analytic gene expression data processing**

Before analysis, gene expression was processed as follow. First, each data set was normalized using Robust Multichip Average (RMA) (7) with the non-parametric quantile algorithm as normalization parameter. Then, we mapped hybridization probes across both platforms. We used NetAffx Annotation files (NetAffx Annalysis Center. http://www.affymetrix.com/analysis/index.affx) to retrieve and update Affymetrix annotations. The probes were then mapped according to their EntrezGeneID. When multiple probes represented the same GeneID, we retained the one with the highest variance in a particular dataset. Normalization was done in R using Bioconductor and associated packages.

**Mutation prediction**

*NPM1* and *FLT3* status were predicted using molecular signatures of respectively 301 and 500 specific probe sets defined by Kholmann *et al*. (8) Classification models were computed using metagene based approach with respective gene lists and statistics for ponderation. With a natural cut-off of 0, samples of each public data set were classified as *NPM1* wildtype- or mutated-*like*. *NPM1*-mut classification using this model was validated with publicly available GEP data (GEO GSE15434) that revealed 94% of correct classification. 188 *NPM1* mutated-*like* patients with survival outcome available were identified from the two cohorts and further analyzed.

***HIST1* mRNA prognosis model**

The 188 CN-AML NPM1-*like* were randomly split into 2 random sets of 94 samples i.e. training and validation set, in which the *HIST1H1D HIST1H2BG* and *HIST1H2BH* 3-genes prognosis model (3-*HIST1*-mRNA) was respectively built and evaluated using Cox regression. We followed the reporting REcommendations for tumour MARKer prognostic studies (9).

**Mass spectrometry analysis.**

Protein extract (5 µg) were loaded on NuPAGE 4-12% Bis-Tris acrylamide gels (Life Technologies) to stack proteins in a single band that was stained with Imperial Blue (Pierce, Rockford, IL) and cut from the gel. Gels pieces were submitted to an in-gel trypsin digestion according to Shevchenko et al^10^. Mass spectrometry analysis were carried out by LC-MS/MS using a Q Exactive Plus mass spectrometer online with a nanoLC Ultimate 3000 chromatography system (Thermo Fisher Scientific™, San Jose, CA). For each biological sample, 25 % of digested sample were injected in duplicate on the system. After pre-concentration and washing on a Acclaim PepMap 100 column (C18, 2 cm × 100 μm i.d. 100 A pore size, 5 μm particle size), peptides were separated on a LC EASY-Spray column (C18, 50 cm × 75 μm i.d., 100 A, 2 µm, 100Å particle size) at a flow rate of 300 nL/min with a linear gradient from 2 to 32% acetonitrile/H20; 0.1 % formic acid for 120 min. The EASYSpray source was set at 1.9 kV and 250 °C. All samples were measured in a data dependent acquisition mode. The peptide masses were measured in a survey full scan (scan range 375-1500 m/z, with 70 K resolution, target AGC value of 3E6 and maximum injection time of 100 ms). Then the 10 most intense data-dependent precursor ions were successively fragmented and measured in Orbitrap (normalized collision energy of 25 %, activation time of 10 ms, target AGC value of 1E3, intensity threshold 1E4 maximum injection time 100 ms, isolation window 2 m/z, 17.5 K resolution, scan range 200 to 2000 m/z). Dynamic exclusion was implemented with a repeat count of 1 and exclusion duration of 20 s.

**Protein identification and quantification**.

**Intensity Based Absolute Quantification (**iBAQ) values was processed using the freely available MaxQuant computational proteomics platform, version 1.5.3.8 (11). iBAQ calculated by MaxQuant are the raw intensities divided by the number of theoretical peptides. Thus, iBAQ values can roughly estimate the relative abundance of the proteins within each sample. This values can be adjusted for each LC-MS/MS by dividing them by the median of the IBAQ values or normalized by dividing them by the sum of iBAQs value in the sample to obtain the molar abundance. The acquired raw LC MS data were first processed using the integrated Andromeda search engine (12). Spectra were searched against a UniProt Human canonical database (version 2017.08; 20201 entries). This database was supplemented with a set of 245 frequently observed contaminants. MaxQuant default parameters were used except mentioned. The match between runs option was enabled to transfer identifications across different LC-MS/MS replicates based on their masses and retention time. The quantification was performed using a minimum ratio count of 1 (unique+razor) and the second peptide option to allow identification of co-fragmented co-eluting peptides with similar masses. The false discovery rate (FDR) at the peptide and protein levels were set to 1% and determined by searching a reverse database. For protein grouping, all proteins that cannot be distinguished based on their identified peptides were assembled into a single entry. The statistical analysis was done with Perseus program (version 1.5.6.0) from the MaxQuant environment (www.maxquant.org). The iBAQ intensities were uploaded from the proteinGroups.txt file. Proteins marked as contaminant, reverse hits, and “only identified by site” were discarded. Proteins list was then export on Excel for further analysis.

**Morphological analyses**

Approximately 10 000 cells were transferred to a slide using a Cytospin centrifuge at 5000 rpm for 5 min at room temperature. Slides were stained at room temperature with May-Grünwald Giemsa (Sigma-Aldrich) and examined for cellular morphology using an structured light ApoTome™ microscope (Zeiss, Münich, Germany) equipped with a 63x 1.4 plan ApoChromat objective and an Axiocam™ MRc5 camera. At least 100 cells per condition were analyzed. For quantification of cells with cytoplasmic granules, at least 100 cells per condition were analyzed and data represent an average of two independent and blind experimenter counts obtained from 3 independent experiments.

**Supplemental references**

1. Bertucci F, Finetti P, Cervera N, et al. Gene expression profiling shows medullary breast cancer is a subgroup of basal breast cancers. *Cancer Res.* 2006;66(9):4636–4644.

2. Irizarry RA, Hobbs B, Collin F, et al. Exploration, normalization, and summaries of high density oligonucleotide array probe level data. *Biostat. Oxf. Engl.* 2003;4(2):249–264.

3. Smyth GK. Linear models and empirical bayes methods for assessing differential expression in microarray experiments. *Stat. Appl. Genet. Mol. Biol.* 2004;3:Article3.

4. Subramanian A, Tamayo P, Mootha VK, et al. Gene set enrichment analysis: a knowledge-based approach for interpreting genome-wide expression profiles. *Proc. Natl. Acad. Sci. U. S. A.* 2005;102(43):15545–15550.

5. Cancer Genome Atlas Research Network. Genomic and epigenomic landscapes of adult de novo acute myeloid leukemia. *N. Engl. J. Med.* 2013;368(22):2059–2074.

6. Metzeler KH, Hummel M, Bloomfield CD, et al. An 86-probe-set gene-expression signature predicts survival in cytogenetically normal acute myeloid leukemia. *Blood*. 2008;112(10):4193–4201.

7. Irizarry RA, Bolstad BM, Collin F, et al. Summaries of Affymetrix GeneChip probe level data. *Nucleic Acids Res.* 2003;31(4):e15.

8. Kohlmann A, Bullinger L, Thiede C, et al. Gene expression profiling in AML with normal karyotype can predict mutations for molecular markers and allows novel insights into perturbed biological pathways. *Leukemia*. 2010;24(6):1216–1220.

9. McShane LM, Altman DG, Sauerbrei W, et al. REporting recommendations for tumor MARKer prognostic studies (REMARK). *Breast Cancer Res. Treat.* 2006;100(2):229–235.

10. Shevchenko A, Wilm M, Vorm O, Mann M. Mass spectrometric sequencing of proteins silver-stained polyacrylamide gels. *Anal. Chem.* 1996;68(5):850–858.

11. Cox J, Mann M. MaxQuant enables high peptide identification rates, individualized p.p.b.-range mass accuracies and proteome-wide protein quantification. *Nat. Biotechnol.* 2008;26(12):1367–1372.

12. Cox J, Neuhauser N, Michalski A, et al. Andromeda: a peptide search engine integrated into the MaxQuant environment. *J. Proteome Res.* 2011;10(4):1794–1805.
